# A comprehensive study on surveillance outcomes of a male population followed at a hereditary breast cancer high-risk consultation at a Portuguese tertiary hospital

**DOI:** 10.1007/s00432-023-04994-7

**Published:** 2023-06-22

**Authors:** Maria João Oliveira, Susy Costa, André Magalhães, Luzia Garrido, Bárbara Peleteiro, José Luís Fougo, Sérgio Castedo

**Affiliations:** 1 Faculdade de Medicina da Universidade do Porto, Alameda Professor Hernâni Monteiro, 4200-319 Porto, Portugal; 2 Surgery Department, Breast Center, Centro Hospitalar Universitário de São João, Porto, Portugal; 3 Medical Genetics Service and Breast Center, Centro Hospitalar Universitário de São João, Porto, Portugal; 4 Institute of Public Health, EPI Unit, University of Porto, Porto, Portugal; 5 Laboratory for Integrative and Translation Research in Population Health, University of Porto, Porto, Portugal; 6 Medical Genetics Service, Centro Hospitalar Universitário de São João, Porto, Portugal; 7 IPATIMUP – Institute of Molecular Pathology and Immunology of the University of Porto, Porto, Portugal; 8 i3S – Institute for Research and Inovation in Health, Porto, Portugal

**Keywords:** Hereditary cancer, Men, Genetic counseling, Risk management, Surveillance, Male breast cancer

## Abstract

**Introduction:**

Men born with pathogenic/likely pathogenic variants in genes associated with the Hereditary Breast and Ovarian Cancer Syndrome have a higher risk to develop breast cancer and other cancers (such as prostate cancer) and should undergo adequate surveillance protocols in highly specialized Centers.

**Methods:**

A retrospective study was conducted to assess these genetic variants’ epidemiological and phenotypical manifestations in male carriers, as well as the efficacy of the surveillance protocol and compliance toward it through a survey. During follow-up, a genetic panel for testing was implemented, the starting age for surveillance was delayed, and the six-month screening interval was extended to annual.

**Results:**

A total of 104 men from a tertiary hospital’s High-Risk Consultation were included, 102 with positive genetic testing for *BRCA1* (*n* = 31), *BRCA2* (*n* = 55), both *BRCA2* and another gene (*n* = 5), *CDH1* (*n* = 2), *CHEK2* (*n* = 4), *NF1* (*n* = 1), *RAD51C* (*n* = 4), and an additional two men with no actionable genetic variant identified. The follow-up period ranged from 1 to 13 years, and only one man developed cancer. Survey responses from 48 men in active surveillance showed that more than half recognizes their carrier status and consequent surveillance impact on their life, including the risk of transmission to offspring, fear of future cancer, meaningful distress, and feeling of injustice. Biannual surveillance was not actively detecting more cancer disease cases, confirming the adequacy of the currently implemented protocol

**Conclusion:**

With support of Genetics to fulfill the current gaps in high-risk management, the proposed redefinition of surveillance protocol would adapt it to the population needs and concerns.

## Introduction

Breast Cancer (BC) is the most frequently diagnosed cancer in women and one of the leading causes of cancer-related death. However, this disease can also appear in men, corresponding to less than 1% of all cases of breast carcinoma and about 0.5% of all malignancies in men in western countries (Campos et al. 2021).

Although it is a rare disease, there has been an increase in the incidence of male breast cancer (MBC) (Gaddam et al. 2021), and it has been shown that survival from BC is significantly lower in men than in women (Abdelwahab Yousef 2017; Liu et al. 2018). All this indicates a natural disadvantage of the male gender facing the disease, strongly explained by the lack of screening recommendations for this population (Gao et al. 2018).

In addition, the lack of consciousness of this entity among the general population means that many men are unaware of the possibility of developing this type of cancer. The field of action on breast pathologies is still very focused on women, causing men to feel socially isolated, stigmatized by their diagnosis, embarrassed in this profoundly feminine environment, and leading to inhibition in searching for information and medical advice (Gethins 2012; Rauscher et al. 2018).

As a result, the diagnosis of MBC is frequently delayed, occurring at advanced stages of disease and, consequently, with a worse prognosis. Due to the lack of robust clinical evidence, surveillance and treatment guidelines for men have been extrapolated from the enormous literature and clinical experience related to BC in women. However, this vast knowledge cannot be linearly adapted to men, who present distinct epidemiological and phenotypic characteristics (Ruddy and Winer 2013).

On the other hand, similarly to what happens in women, genetic predisposition is a significant risk factor in men. Despite its low incidence in the general population, men born with genetic variants associated with hereditary BC have a higher risk of developing it, as well as other cancers (such as prostate cancer (PC)). *BRCA1* and *BRCA2* are the most frequently involved genes; however, thanks to the widespread use of the Next-Generation Sequencing (NGS) technique, other genes have been identified as possible contributors to the disease, such as *PALB2*.

Carriers of pathogenic and likely pathogenic (P/LP) variants in these genes undergo surveillance protocols, and, to the best of our knowledge, in most cases, the development of cancer disease is rare. In this context, it is questionable whether the surveillance proposed for these asymptomatic carriers is the most appropriate, considering the psychosocioeconomic impact that cannot be underestimated, validated by the scarcity of personal and material resources, and also marked by a significant embarrassment that could be avoided (Freitas et al. 2018; Rauscher et al. 2018).

This study aims to assess how P/LP variants in genes associated with Hereditary Breast and Ovarian Cancer Syndrome (HBOCS) manifest epidemiologically and phenotypically in male carriers as well as the efficacy of the surveillance protocol. The compliance toward it was also evaluated by collecting the at-risk individuals’ opinions.

## Methods

A retrospective review was performed on male individuals carrying demonstrably identified (positive molecular study and, in asymptomatic carriers, positive counter analysis) variants in genes associated with hereditary BC, followed between January 2008 and April 2022 in the Oncogenetics and the High-Risk Consultations of the Breast Center of Centro Hospitalar Universitário de São João (CHUSJ). The molecular study immediately provided variants’ pathogenicity, and ClinVar database was used to search for recent reclassification of variants of uncertain significance (VUS).

By a retrospective analysis of the clinical records, demographic and epidemiological information was retrieved, including age, marital status, number of children, smoking habits, comorbidities, follow-up start date, genetic variant identified, and family history of cancer.

Until 2016, molecular testing consisted in this study of a singular gene at a time. After that year, the Oncogenetic Department of CHUSJ started providing the NGS breast-specific panel, which simultaneously analyzed five–six genes by then and nowadays ~ 20 genes related to BC.

To manage the genetic risk, every carrier had one first consultation to provide proper counseling and discuss the possibility of preimplantation genetic diagnosis (PGD). Then, at-risk individuals were submitted to the surveillance protocol proposed by the National Comprehensive Cancer Network (NCCN) guidelines in force at that time: recommendation of clinical breast examination, digital rectal examination, breast imaging, and PSA serum measurement every six months, starting at 35 years old. In 2016, the High-Risk Consultation medical team extended this period to annual, based on NCCN update guidelines version 2.2015, and began recommending surveillance starting at 40 years old.

Considering these changes, there was interest in studying which differences would arise and which would be men’s behavior toward them.

Among the subset of male carriers diagnosed with cancer, we evaluated the date of diagnosis and whether it had been prior to or after the genetic study, primary and secondary tumors, anatomopathological stage according to the TNM classification system, treatment performed, and health condition at last observation.

To complement the investigation, a survey was conducted via telephone to men ≥ 40 years old in active surveillance for at least 2 years to assess the psychosocioeconomic impact of their carrier status and compliance with the protocol. In the absence of a known validated survey aimed at assessing this impact, we elaborated one, with questions directed at evaluating the emotional implications of their carrier status, interference in work and social activities, and satisfaction with the current surveillance protocol. It was performed in a semi-structured format, asking participants to classify statements according to the 5-point Likert scale and allowing them to freely express their opinions on the topic. The purpose of this survey was explained to participants at first, predicting tacit informed consent when answering the questions.

Data analysis was subsequently performed using IBM SPSS version 28.0.1.0 statistical software, presenting absolute and relative frequency for categorical variables and the median and interquartile range for continuous variables.

## Results

### Demographics

A total of 104 men followed in the High-Risk Consultation were included in our study. Personal history of cancer (non-HBOCS) was present in 7.7%, 50% of the carriers had no comorbidities, 31.7% had cardiovascular risk factors or cardiopulmonary disease, and 10.7% had other comorbidities (infectious, inflammatory, hepatobiliary, intestinal, musculoskeletal, gastric, or renal). A detailed overview of the participants’ characteristics is presented in Table 1.

**Table 1:** Participant’s characteristics

	Total [*n* = 104] *N* (%)
*Age at first High-Risk Consultation*, years [median (range)]	43.5 (19–75)
*Marital status*^a^	
Married/civil union	77 (74.0)
Single/divorced	24 (23.1)
*Children*^b^	
No	38 (36.5)
Yes	64 (61.5)
*Smoking habits*^c^	
Non-smoker	60 (57.7)
Current/former smoker	38 (36.5)
*Comorbidities*	
No comorbidities	52 (50.0)
Cardiovascular risk factor or cardiopulmonary disease	33 (31.7)
Cancer	8 (7.7)
Others	11 (10.7)
*Age at genetic testing*, years [median (range)]	43.0 (18–75)
*Carrier and clinical status*	
Symptomatic carrier	9 (8.7)
Index case	4 (3.9)
Relative	5 (4.8)
Cancer diagnosis prior the genetic testing	3 (2.9)
Cancer diagnosis after the genetic testing	2 (1.9)
Asymptomatic carrier	93 (89.4)
No actionable variant identified	2 (1.9)
Symptomatic	1 (0.9)
Asymptomatic	1 (0.9)
*Altered gene*	
* BRCA1*	31 (29.8)
* BRCA2*	55 (52.9)
* BRCA1* + *BRCA2*	1 (1.0)
* BRCA2* + *ATM*	2 (1.9)
* BRCA2* + *CDH1*	1 (1.0)
* BRCA2* + *MSH6*	1 (1.0)
* CHEK2*	4 (3.8)
* RAD51C*	4 (3.8)
* CDH1*	2 (1.9)
* NF1*	1 (1.0)
No actionable variant identified^d^	2 (1.9)
*Genetic variant*	
Pathogenic	90 (86.5)
Likely pathogenic	6 (5.8)
VUS^e^	6 (5.8)
No actionable variant identified	2 (1.9)
*Follow-up time*, years [median (range)]	5 (1–13)
*Lost to follow-up*	7 (6.7)
*Dead*	3 (2.9)

^a^Unknown for 3 participants

^b^Unknown for 2 participants

^c^Unknown for 6 participants

^d^Genetic testing not performed in 1 participant

^e^Variant of Uncertain Significance

Of the 104 participants, 102 were followed because of their carrier status, one with BC but without a causative variant identified and the other one with a strong family history of cancer but no causative variant identified in the affected family members. According to this, they were classified as symptomatic carriers with an actionable variant identified (*n* = 9, 8.7%), asymptomatic carriers with an actionable variant identified (*n* = 93, 89.4%), and men with no actionable variant identified (*n* = 2, 1.9%), including one symptomatic and one asymptomatic (Table 1). Four of the nine symptomatic carriers were studied as index cases. The remaining five were relatives—three had their cancer diagnosed previously, and the other two after genetic testing.

### Genetic testing

The main criteria for testing were a known hereditary cancer P/LP variant in the family or a male proband with the diagnosis of BC, with a median age at the time of testing of 43 years old (ranging from 18 to 75). Only one man (1.0%) from the study population had not performed genetic test, as a *BRCA2* VUS has been identified in the index case (brother who had BC), and there is no indication to search for VUS in healthy individuals (NCCN, 2023), although the High-Risk Consultation medical team decided to continue the surveillance program based on his family history of cancer. Of the 103 males tested, 6 (5.8%) were index cases, three of which were studied before 2016 and the other 3 after. The remaining 97 (94.2%) males were family members, who were only checked for the specific family variant previously identified in the index case (Table 1).

A total of 55 men (52.9%) were positive for *BRCA2* variants, 29.8% (*n* = 31) for *BRCA1*, and 4.9% (*n* = 5) for both *BRCA2* and another gene. Variants in non-*BRCA* genes were identified in 10.5% (*n* = 11) of the men (Table 1). Before 2016, genetic alterations were identified in *BRCA1* (*n* = 6, 5.8%), *BRCA2* (*n* = 26, 25.0%), and *CDH1* (*n* = 1, 1.0%), while, after 2016, genetic variants in *BRCA1* (*n* = 25, 24.0%), *BRCA2* (*n* = 29, 27.9%), *BRCA1* + *BRCA2* (*n* = 1, 1.0%), *BRCA2* + *ATM* (*n* = 2, 1.9%), *BRCA2* + *CDH1* (*n* = 1, 1.0%), *BRCA2* + *MSH6* (*n* = 1, 1.0%), *CDH1* (*n* = 1, 1.0%), *CHEK2* (*n* = 4, 3.8%), *NF1* (*n* = 1, 1.0%), and *RAD51C* (*n* = 4, 3.8%) were identified. The classification of the identified genetic variants as pathogenic, likely pathogenic or VUS is described in Table 1. In one man (1.0%) from the study population, the genetic test was negative for *BRCA2* gene alterations.

### Surveillance

Men not old enough to start active surveillance joined the non-eligible group for surveillance and were discharged from the High-Risk Consultation until they reached the required age (initially 35 and then 40 years old).

The follow-up period ranged from 1 to 13 years, with a median period of 5 years. Two eligible men did not get to start the surveillance program, since one of them decided to proceed with risk management in his hometown region, and the other one was soon referred to Palliative Care due to the progression of his PC. Seven were lost to follow-up (Table 1).

### Cancer characteristics

Of the 104 participants, 10 (9.6%) presented with cancer disease. MBC (*n* = 3) was the most diagnosed cancer, followed by PC (*n* = 2) and gastric cancer (*n* = 2). Other cancers found in this group included choroidal melanoma (*n* = 1), glioblastoma (*n* = 1), pheochromocytoma (*n* = 1), GIST (*n* = 1), and colorectal cancer (*n* = 1). The underlying genotypic and phenotypic characteristics of each symptomatic case are summarized in Table 2.

**Table 2:** Symptomatic patients’ characteristics [*n* = 10]

Patient	Age at first High-Risk Consultation, years	Marital status, number of children	Smoking habits Comorbidities	Family history	Cancer type	Tumor staging	Treatments performed	Age at cancer diagnosis, years Timing in relation to surveillance	Age at molecular diagnosis, years	Altered gene Genetic variant	Follow-up time, years	Relapse, years from initial diagnosis	Vital status
A	63	Married, 1	Non-smoker No comorbidities	2 sisters with breast cancer (diagnosed at 50 and 65 years old)	Luminal IDC in left breast with vascular invasion and focal nipple ulceration	pT4N0M0	CT, RT, HT, Mastectomy	60 Previous	63	*BRCA2* c.156_157insAlu in heterozygosity Pathogenic	8	No	Alive
B	62	Married, 2	Former smoker No comorbidities	Paternal grandmother with breast cancer (died at 82 years old)	IDC in left breast	pT1cN1miM0	CT, RT, HT, Mastectomy	59 Previous	61	*BRCA2* c.608C > A p.(Thr203Asn) in heterozygosity VUS	6	No	Alive
C	62	Married, 4	No information No comorbidities	Absent	Intermediate-grade DCIS in the right breast	pTisN0M0	Mastectomy	62 Previous	63		8	No	Alive
D	46	Single, 0	Current smoker No comorbidities	Mother with breast cancer (diagnosed at 33 years old)	Prostate adenocarcinoma Gleason 8 (4 + 4) with perineural invasion and disseminated bone metastasis	T1cNXM1b		44 Previous	45	*BRCA2* c.156_157insAlu in heterozygosity Pathogenic	Not performed		Dead
E	45	Married, 1	Current smoker No comorbidities	2 brothers with gastric cancer (diagnosed at 23 and 27 years old)	Diffuse gastric cancer	T1N0M0	Prophylactic total gastrectomy	34 Previous	48	*CDH1* c.1901C > T p.(Ala634Val) in heterozygosity Likely pathogenic	5	No	Alive
F	36	Civil union, 2	Current smoker No comorbidities	Beyond 3^rd^grade relatives	Diffuse gastric cancer	pT2N0M0	Therapeutic gastrectomy	35 Previous	33	*CDH1* c.1901C > T p.(Ala634Val) in heterozygosity Likely pathogenic	3	No	Alive
G	64	Married, 1	Non-smoker No comorbidities	Mother and half-brother with breast cancer (diagnosed respectively at 52 and 57 years old)	Colorectal cancer	Missing		46 Previous	64	*BRCA2* + *MSH6* c.156_157insAlu in heterozygosity + *MSH6* VUS Pathogenic	1	No	Alive
H	33	Married, 1	Non-smoker Cancer (pheochromocytoma, multiple jejunal GIST)	Mother with cutaneous neurofibromas (diagnosed at 57 years old)	Pheochromocytoma, multiple GIST in the small bowel	Missing	Adrenalectomy, Jejunal segmental enterectomy	32 Previous	33	*NF1* c.6789_6792del (p.Tyr2264Thrfs*5) in heterozygosity Pathogenic	Not eligible		Alive
I	67	Married, 4	Non-smoker Cardiovascular risk factor (arterial hypertension)	Daughter with breast cancer (diagnosed at 32 years old)	Prostate adenocarcinoma Gleason 9 (4 + 5) with biochemical relapse	pT2N0M0	Radical prostatectomy, salvation radiation	67 After	65	*BRCA2* c.156_157insAlu in heterozygosity Pathogenic	12	Yes, 5	Alive
J	72	Married, 1	Non-smoker Cardiovascular risk factors (arterial hypertension, dyslipidemia, coronary disease)	Daughter with breast cancer (diagnosed at 42 years old)	In situ choroidal melanoma in right eye, glioblastoma IDH wild type	Missing	CT, RT, Microsurgical removal of the brain tumor	77 After	72	*BRCA2* c.156_157insAlu in heterozygosity Pathogenic	6	No	Dead

*IDC* Invasive Ductal Carcinoma, *DCIS* Ductal Carcinoma in situ, *CT* Chemotherapy, *RT* Radiotherapy, *HT* Hormonotherapy

At the time of genetic testing, eight patients were already diagnosed with cancer (Patients A to H). The surveillance protocol allowed the detection of only one PC case (Patient I), diagnosed in advanced stage in 2010, throughout the investigation performed over imaging findings recorded in his first examination.

### Survey

**Fig. 1:**
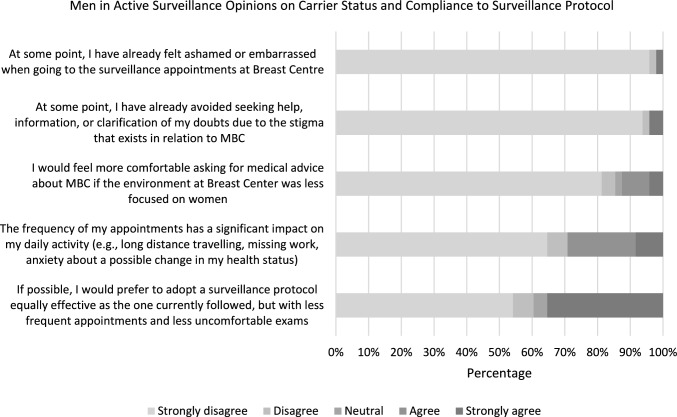
Men in active surveillance opinions on carrier status and compliance to surveillance protocol (*n* = 48)

The survey was completed by all 48 invited men, with a median age of 60 years old (ranging from 42 to 79). Figure 1 summarizes the answers obtained to the corresponding questions.

Overall, the participants showed no significant embarrassment going to the surveillance consultations nor looking for information about MBC, even referring to feel comfortable at the Breast Center despite its feminine environment.

About 70% of inquired men denied inconveniences attending the appointments. On the contrary, about 30% (most of them in active working age) showed a willingness to extend the screening interval and preference for less uncomfortable exams, highlighting the psychosocioeconomic impact of undergoing surveillance to manage their hereditary cancer risk.

## Discussion

This study examined 102 men with P/LP variants associated with hereditary cancer and 2 without any actionable variant identified. Most of these men had no significant comorbidities, although a third had cardiovascular risk factors or cardiopulmonary disease. At the time of genetic testing, P/LP variants and VUS were primarily identified in *BRCA1*/*2* (87.5%) but also in non-*BRCA* genes (14.4%). Ten participants presented with cancer (MBC being the most common and PC the second most), with only one case (1.0%) detected during the follow-up period due to screening. Most participants did not have significant apprehension about attending surveillance appointments. However, some would prefer less uncomfortable exams and longer screening intervals, demonstrating the psychosocioeconomic impact associated with surveillance.

The prevalence of *BRCA1* and *BRCA2* genetic variants in the general population is estimated to be between 1 in 500 and 1 in 1000, respectively (Freitas et al. 2018). Despite these low rates, P/LP variants in these genes are the most frequent genetic alterations diagnosed in familial BC, which aligns with our study population. Other moderate penetrance genes have also been described as possible contributors to hereditary BC, associated with an average age at MBC onset lower than without any P/LP variants but also lower than with *BRCA1/2* P/LP variants (Tedaldi et al. 2020). Multigene panel testing, as a regular practice, will likely continue to detect germline variants in other non-*BRCA1*/*2* genes, which are individually rare but confer a modest increase in cancer risk with questionable associated clinical significance (Freitas et al. 2018; Katona et al. 2018). Our study confirmed this by detecting *ATM*, *CDH1*, *MSH6*, *CHEK2*, *NF1*, and *RAD51C* genetic variants through NGS.

Some MBC series have shown that *ATM* and *PALB2* are the most frequent non-*BRCA* genes affected by P/LP variants. Previous studies have reported a 1% BC risk and a 3% pancreatic cancer risk for men carrying *PALB2* variants by age 80 (Tedaldi et al. 2020), underlying its relevant role in MBC predisposition and the importance of developing a surveillance protocol for male carriers. On the other hand, the involvement of the *ATM* gene in the predisposition to the disease is very scarce and should be further investigated in more extensive case series (Campos et al. 2021; Rizzolo et al. 2019), as in agreement with the only *ATM* genetic variant carrier in our study population, who had not developed MBC.

Genetic variants in the *CHEK2* gene have been associated with an increased risk of breast, prostate, and other cancers (Southey et al. 2016; Wu et al. 2018). In fact, the *CHEK2* 1100delC variant may explain up to 5% of BC families with a *BRCA1/2* phenotype but with a *BRCA1/2*-negative test result, which has been shown to increase MBC risk by tenfold (Freitas et al. 2018; Tedaldi et al. 2020). This information is particularly relevant to Patient C—having a personal history suggestive of an inherited susceptibility and a *BRCA2*-negative genetic test performed in 2008 (when it was only searched for *BRCA2* genetic variants), it is our understanding that this man should be invited for retesting, according to NCCN (2023) recommendations, incorporating these recently MBC-associated genetic alterations, namely, the *CHEK2* 1100delC variant.

*CDH1* pathogenic variants appear only to be associated with lobular breast cancer and not ductal breast cancer or other rare types of breast cancer. Since lobular development does not occur in the male breast, men carrying *CDH1* P/LP variants are at no increased risk of developing BC (Blair et al. 2020; Carnevali et al. 2022). That is why, nowadays, these men do not undergo surveillance for BC.

*RAD51C* and *RAD51D* germline alterations have been associated with increased ovarian cancer risk, whereas their contribution to BC risk is less clear (Clague et al. 2011; Silvestri et al. 2011; Tedaldi et al. 2020). Due to the low frequency of *RAD51C* MBC, there is currently insufficient evidence to recommend breast cancer screening in male carriers of these variants (NCCN 2023).

The relationship between the neurofibromatosis type 1 gene and BC in women is known; by contrast, the current presentation of the *NF1* gene and BC in men is a rare phenomenon (Rizzolo et al. 2019). Likewise, it is not currently considered for MBC risk management at the High-Risk Consultation of our Breast Center.

In our population, no P/LP variants were found in the *TP53* gene. Pathogenic variants in this gene have been reported among women with BC, while it may not play a significant role in MBC (Rizzolo et al. 2019). These patients are followed up at the Medical Oncology High-Risk Consultation because of their risk to develop multiple cancers.

Finding a non-classified VUS motivates the analysis of the familiar phenotype to infer its degree of pathogenicity (NCCN 2023). The one man in our study who had not performed genetic testing belongs to a family that would have been on hold in terms of investigation for several years. Since, nowadays, it is proven there are other genes associated with MBC risk, there is an indication to extend the genetic testing in the index case, with obvious implications for himself and his family, if an actionable variant is identified in one of the non-*BRCA* genes. If a positive result is obtained, it will be beneficial to search for the same alteration in his brother.

The only cancer detected during follow-up occurred in 2010, before the periodicity change in surveillance protocol. With the greater spacing of consultations from 2016, the fear was that more interval cancers would appear, yet it did not happen. This means that, even though in the first phase of our study patients were monitored biannually, a 6-months interval surveillance was not actively detecting more cases of cancer disease, confirming the adequacy of the NCCN protocol currently implemented—although, as practiced in this study’s follow-up period since 2016, we consider it is appropriate starting surveillance at 40 years old [instead of the 35 years proposed by NCCN (2023)].

Nowadays, even with annual surveillance, patients are sometimes forced to go to the hospital more often than strictly necessary. Due to the widespread use of imaging techniques for more precise diagnosis, there is a consequent difficulty in scheduling and processing them in a timely manner, which significantly impacts patients’ daily activities. Besides, when it comes to favorable surveillance outcomes, the unnecessary expenses of human and material resources must be a matter of concern when improving the pathway of care.

Because of the small number of men with cancer disease, there were insufficient data for a meaningful outcome analysis.

As expected, *BRCA1/2* pathogenic variants were the most frequent genetic variants found between symptomatic carriers. Men with *BRCA1* variants have a lifetime breast cancer risk of 1–5%, 2–3% of pancreatic cancer, and 7–26% risk of PC. Altered *BRCA2* carriers have a 5–10% lifetime breast cancer risk, 15–61% risk of PC, 3–5% risk of pancreatic cancer, and 3–5% risk of melanoma (Rauscher et al. 2018).

Up to 14% of men diagnosed with BC are found to harbor a *BRCA2* variant (Ibrahim et al. 2018), which is in line with the MBC cases in our population—two in three MBC cases presented a *BRCA2* variant, confirming its role as the key gene associated with increased risk of developing this type of cancer. Evidence suggests MBC is associated with an elevated risk of second tumors other than BC (Campos et al. 2021); however, no men with BC from our population presented with secondary cancers.

The risk of PC is up to five-fold higher in *BRCA2* variant carriers than in general population and has been reported to be more aggressive (frequently with Gleason scores ≥ 8) and associated with worse survival compared to *BRCA* wild-type cancers (Ibrahim et al. 2018). As observed in our study, the two patients with PC had the same *BRCA2* pathogenic variant, one (Patient D) with Gleason 8 and bone metastasis at presentation and the other one (Patient I) with biochemical relapse treated with salvation radiation.

Emerging data have suggested a potential benefit of pancreatic cancer and melanoma screening in selected individuals at increased risk. Because of limited definite data on long-term screening results, each situation should be evaluated based on family history and jointly decided with the patient (Campos et al. 2021; Silvestri et al. 2020; Tedaldi et al. 2020). According to NCCN (2023), studies investigating *BRCA2* P/LP variants and melanoma showed some evidence of an association, even though inconsistent conclusions have been drawn. The development of in situ choroidal melanoma (1.0%) in Patient J (positive for a *BRCA2* pathogenic variant) reinforces the need for subsequent studies on this possible association.

One of the questions that remain to be answered is the management of high-risk patients with VUS. About 5.8% of our male referrals to our High-Risk Consultation had an inconclusive genetic result. This can be a source of distress for patients and their families and poses a unique problem in risk management (Freitas et al. 2018), but, since Patient B carries a *BRCA2* VUS and developed MBC, our study demonstrates the urge for further investigations in order to clarify the impact of these variants, reclassify them, and fulfill the lack of predictive cancer models for their carriers.

As demonstrated, few men with P/LP variants in genes associated with HBOCS developed MBC and PC. The incidence of MBC varies widely across geographical areas, and country-specific environmental influences and lifestyle factors cannot be excluded (Abdelwahab Yousef 2017; Silvestri et al. 2020). Besides genetic contribution, we investigated the susceptibility to MBC by analyzing other possible risk factors, like smoking habits, comorbidities, and family history of cancer. Contrary to other studies (Gucalp et al. 2019; Liu et al. 2018), our findings demonstrate that, in our population, none seemed to have an evident influence on cancer development.

Emerging literature suggests that many clinicians make inappropriately aggressive management recommendations for individuals carrying P/LP variants, and increased surveillance of healthy *BRCA1*/*2* male carriers is still controversial (Freitas et al. 2018). Even if higher cancer risk is recognized, the distress and psychological needs of male patients getting genetic testing and counseling cannot be ignored to improve the pathway of care (Campos et al. 2021; Katona et al. 2018).

Survey responses showed that, in almost half of the inquired men, the identification of a P/LP variant with associated risk to MBC or PC did not change anything on a daily basis. In fact, previous researches show that men from HBOCS families actively seek counseling and are usually compliant with increased surveillance (Freitas et al. 2018). However, more than half of the survey population recognizes that the identification of a genetic alteration has impact on their life. As described in the literature, one-third of the men in this inquiry pointed to the transmission of their genetic predisposition to their children as one of the biggest concerns related to their carrier status (van der Post et al. 2015). This reinforces the potential benefit of reproductive counseling and PGD for at-risk couples, a technique that allows embryos to be tested before the transfer to the uterus, pursuing implantation according to their genetic variant carrier status (Freitas et al. 2018; van der Post et al. 2015). Across participants, nine individuals referred being afraid of developing cancer in the future, especially when they had a strong family history of cancer, and other two reported meaningful distress and feeling of injustice toward the situation. Also, four carriers became more alert after the positive genetic testing, yet without greater anxiety due to the trust in the High-Risk Consultation medical team. Respondents emphasized that this comes across more specialized training and monitoring experienced at highly specialized Centers, which reflects the current conditions of Medical Genetics practice in Portugal. The historical allocation of genetic services to tertiary care results in geographical, financial, and psychological barriers (Costa et al. 2022). However, general and family medicine specialists are the first medical point of contact within the health system. Training and education in genetics should be integrated into primary care, which could guarantee appropriate referrals and timely diagnosis from a perspective closer to the individual and his surroundings.

Study limitations include its retrospective nature. However, given the long period of follow-up (12 years), it was possible to reach a large number of participants in our study. Our population was recruited from a tertiary ERN GENTURIS health care provider institution in a dedicated center certified by EUSOMA. In fact, the High-Risk Consultations were always performed by the same three medical care providers, following the same guidelines (NCCN). The second limitation is the small amount of novel MBC-associated genes carriers that precludes clinical information from being totally informative in elucidating the clinical phenotype associated with them. Regarding the uncommon nature of MBC, although we cannot draw robust conclusions, these data serve as a point of reference for national averages for many of the behavioral variables discussed. Another limitation is having a high percentage (32.7%) of asymptomatic carriers under 40 years old who are not yet eligible to start surveillance—although they were included in our sample, they are not helpful yet in retrieving important outcome information to support our conclusions. As strength of this study, we highlight very little research investigating the effective impact of P/LP variants associated with hereditary MBC and considering the implications of the surveillance itself from a holistic point of view of the patient.

## Conclusions

To the best of our knowledge, no studies have taken simultaneously into account all the issues considered in our study, using a survey to describe the effects of breast and prostate surveillance on high-risk men’s quality of life.

These data may represent a step toward evidence-based guidelines specific for men with P/LP variants in genes associated with hereditary breast cancer syndromes. As surveillance protocol, we suggest clinical breast examination, digital rectal examination, breast imaging, and PSA serum measurement every 2 years starting at 40 years old (or 10 years before the earliest known MBC or PC case in the family) and annually from 50 years old beyond. Even though further national studies are needed to corroborate our results, this proposal could adapt the risk management to the national reality and, especially, to the population needs, lowering its psychosocioeconomic impact and most likely without an increase in the incidence of cancer.

As important as screening at-risk men and diagnosing cancers in early stages, it is crucial to support and accompany these carriers who are going through a situation of uncertainty inherent to the carrier status itself and the still insufficient knowledge about HBOCS in men. Also, considering everything analyzed in this study, when referring exclusively to men, instead of designating HBOCS, we propose the designation of Hereditary Breast and Prostate Cancer Syndrome (HBPCS).

Therefore, there is an urgent need to improve genetic education and training and create special units for specific diseases, as well as a better organization and composition of services in a multidisciplinary and user-centered approach, fulfilling the current gaps concerning the optimal management of high-risk individuals.

## Data Availability

Data are not freely available as genetic data are sensitive data, for which we are obliged legally and ethically in our country to restrict the access due to preserving patient privacy.
